# AMIDE v2: High-Throughput Screening Based on AutoDock-GPU and Improved Workflow Leading to Better Performance and Reliability

**DOI:** 10.3390/ijms22147489

**Published:** 2021-07-13

**Authors:** Pierre Darme, Manuel Dauchez, Arnaud Renard, Laurence Voutquenne-Nazabadioko, Dominique Aubert, Sandie Escotte-Binet, Jean-Hugues Renault, Isabelle Villena, Luiz-Angelo Steffenel, Stéphanie Baud

**Affiliations:** 1 Université de Reims Champagne Ardenne, ESCAPE EA 7510, 51097 Reims, France; pierre.darme@univ-reims.fr (P.D.); daubert@chu-reims.fr (D.A.); sandie.escotte@univ-reims.fr (S.E.-B.); ivillena@chu-reims.fr (I.V.); 2 Université de Reims Champagne Ardenne, CNRS, ICMR UMR 7312, 51097 Reims, France; laurence.voutquenne@univ-reims.fr (L.V.-N.); jean-hugues.renault@univ-reims.fr (J.-H.R.); 3 Université de Reims Champagne Ardenne, CNRS UMR 7369, MEDyC, 51097 Reims, France; manuel.dauchez@univ-reims.fr; 4 Plateau de Modélisation Moléculaire Multi-échelle, URCA, 51097 Reims, France; arnaud.renard@univ-reims.fr (A.R.); luiz-angelo.steffenel@univ-reims.fr (L.-A.S.); 5 Université de Reims Champagne Ardenne, LICIIS–LRC CEA DIGIT, 51097 Reims, France; 6 Centre Nationale de Référence de la Toxoplasmose, CHU Reims, 51092 Reims, France

**Keywords:** molecular docking, screening, AutoDock, parallelization, GPU, high performance computing, *Toxoplasma gondii*

## Abstract

Molecular docking is widely used in computed drug discovery and biological target identification, but getting fast results can be tedious and often requires supercomputing solutions. AMIDE stands for AutoMated Inverse Docking Engine. It was initially developed in 2014 to perform inverse docking on High Performance Computing. AMIDE version 2 brings substantial speed-up improvement by using AutoDock-GPU and by pulling a total revision of programming workflow, leading to better performances, easier use, bug corrections, parallelization improvements and PC/HPC compatibility. In addition to inverse docking, AMIDE is now an optimized tool capable of high throughput inverse screening. For instance, AMIDE version 2 allows acceleration of the docking up to 12.4 times for 100 runs of AutoDock compared to version 1, without significant changes in docking poses. The reverse docking of a ligand on 87 proteins takes only 23 min on 1 GPU (Graphics Processing Unit), while version 1 required 300 cores to reach the same execution time. Moreover, we have shown an exponential acceleration of the computation time as a function of the number of GPUs used, allowing a significant reduction of the duration of the inverse docking process on large datasets.

## 1. Introduction

Molecular docking consists of modelling the interaction between two molecules at the atomic level. The primary process is to predict the position and the orientation of a small molecule (ligand) relative to a biological target (receptor) and consequently their affinity. The quality of the resulting interaction can then be used to hypothesize the mechanism driving the formation of the complex. In this way, molecular docking can highlight the potential biological activity of a compound on a specific target, and more widely on an organism. 

Docking can be used in several ways. The first is simple docking (one ligand, one target) [1] to qualify and quantify the interaction between a small molecule and a known protein in order to confirm or explain a biological activity on a specific target. The second one is known as virtual screening (multiple ligands, one target) [2]. This consists of selecting the small molecules with the best predicted affinity for a known protein and identifying the best complex. Such in silico protocol helps to characterize biological processes such as the inhibition of a specific metabolic pathway. The third one is inverse virtual screening (one ligand, multiple targets) [3]. The latter can be used in high-throughput screening workflow to find hits for a chemical structure extracted from a chemical library and a protein dataset [4].

The list of docking software is comprehensive and has grown during the last decades. Some are open source (AutoDock, CABS-dock, FlexAID) [5,6,7], others are commercial (Glide, GOLD, Surflex-Dock) [8,9,10] or are even linked to a web service (Click Docking, AADS, DockingServer) [11,12,13]. They are generally suitable for classical docking, but they are not designed for high throughput in silico screening, for instance, they involve time-consuming and tedious manual steps for docking parameter selection. Therefore, some projects exist to alleviate indicated counterparts, such as VirtualFlow orchestrator, which is based on AutoDock 4.2 docking engine [14]. Nevertheless, this software still does not support the newly released AutoDock-GPU [15]. Finally, even if the literature attests to the development of many frameworks, most are in-house developed and not available online.

Inverse docking is a powerful tool in target discovery. However, since conventional tools are mainly developed for simple docking or screening and do not directly allow multiple receptors, most available tools suffer from a lack of speed in execution time and thus prevent fast identification of relevant biological targets and pharmacological agents. Time efficiency becomes a major issue in emergency situations such as searching for drugs or vaccines against pandemic diseases (malaria, AIDS or SARS-CoV-2, for example).

AMIDE (AutoMated Inverse Docking Engine) [16] was initially reported in 2014. This inverse virtual screening tool was based on AutoDock 4.2 [5]. The purpose was to bypass the limitations presented above for large-scale molecular docking by automating many tasks and optimizing the use of Information Technology (IT) resources. It was compatible with conventional computers, but its use was more efficient on HPC (High-Performance Computing) platforms. AutoDock 4.2 (autodock and autogrid) was initially chosen for the primary development of AMIDE (named AMIDE v1 in this work) because it is in constant development, and its reliability has been extensively tested and validated by the scientific community [17]. It is also easy to access and highly configurable. AutoDock 4 allows evaluation of the free energy of binding and accelerates the search of relevant solutions through Lamarckian Genetic Algorithms [5]. This software is described as giving a fast prediction of bound conformations and free energy of binding based on molecular mechanics equation added of entropic terms [18] as shown by Equation (1):

(1) $$\Delta G=\Delta G_{vdw}+\Delta G_{hbond}+\Delta G_{elec}+\Delta G_{conform}+\Delta G_{tor}+\Delta G_{sol}$$

Equation (1): Main equation of AutoDock 4 defining the free energy of binding. The terms Δ*G_vdw_*, Δ*G_hbond_*, Δ*G_elec_* and Δ*G_conform_* are molecular mechanics terms (respectively representing van der Waals, hydrogen bonds, electrostatic and conformational energies) while Δ*G_tor_* and Δ*G_sol_* are AutoDock 4 added terms representing torsional and desolvation energies.

**Figure 1:**
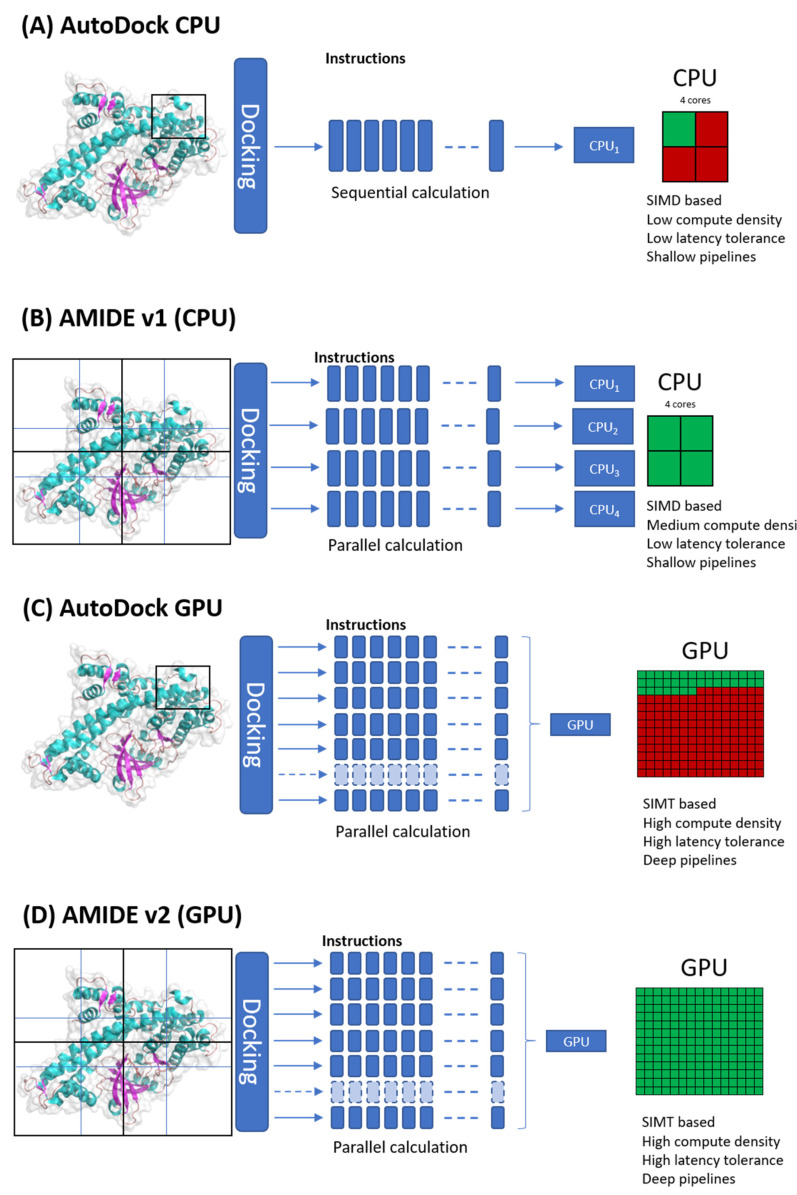
Comparison of AutoDock 4.2 (CPU), AutoDock-GPU and the two versions of AMIDE framework. While AutoDock standalone software can only dock one manually defined box at once (**A**), the AMIDE protocol automatically cuts the receptor into preselected overlapping boxes. AMIDE v1 boxes were independently sent to all available CPU cores (**B**). AutoDock-GPU is based on embarrassingly parallel capacities and can obtain multiple docking calculations in input (**C**). AMIDE v2 is now using GPU based parallelization, allowing time calculation decrease by computing multiple dockings at once (**D**).

Whereas the classical use of AutoDock needs the computation of a single pre-processing grid, the AMIDE strategy consists of cutting the tridimensional receptor structures into smaller grids. The grids are overlapping so that no information is lost through unexplored regions. A slicing in 8 (2 × 2 × 2), 12 (3 × 2 × 2), or 27 (3 × 3 × 3) boxes is possible, but the 12 overlapping boxes slicing showed optimal docking quality [16]. All the sub-docking experiments are then simultaneously performed independently of each other thanks to CPU (Central Processing Unit) parallelization. The calculation time is reduced since the calculation is not batched on the single-threading instance but rather separated into multicore processes. This distribution depends on the number of processing cores or even on the number of processors, as shown in Figure 1.

However, this workflow leads to some limitations. Since AutoDock 4.2 was developed for a CPU architecture, a docking job is performed on a single core, which implies the use of several cores to obtain the expected performance. One side effect is the difficulty to obtain a relevant speed calculation in personal computer, while the best approach is to use HPC architecture. Besides, the demand for numerous cores increases the data flow, eventually leading to input/output errors. Some errors are not intrinsically caused by AMIDE v1 and are hardware or software dependent (for instance, global hardware capacity or workload manager user limits).

Among the main drawbacks associated with AMIDE v1, the fact that slicing and grids generation were performed for each couple of ligand and receptor led to considerable time elongation even with grid generation parallelization. In addition, AMIDE v1 was only able to perform inverse docking of one ligand at a time, which made tedious its use for High-Throughput inverse docking (multiple ligands, multiple proteins). This limitation could be bypassed by manual intervention at the end of each ligand inverse docking, but was still not competitive for large ligands and receptors databases screening.

A new version, called AMIDE v2, was thus developed to speed up the inverse docking process. This framework is now based on AutoDock-GPU [15] and has benefited from the total revision of the programming workflow.

The advent of a new version of AutoDock based on calculation parallelization using GPUs (Graphics Processing Units) was the start of AMIDE v2. The performance gains were intended to be considerable. As previously mentioned, several front-end modifications to AMIDE were made beyond the use of this latest version of AutoDock. All these improvements led to better performances and reliability in inverse docking using AMIDE. This new and optimized workflow can handle multiple ligands, and AMIDE is now adapted to large scale double docking, i.e., multiple ligands on multiple targets simultaneously.

The updated AMIDE v2 version, whose performances are demonstrated in the present paper, was applied to a test case mimicking the identification of new chemical structures to tackle *Toxoplasma gondii* (*T. gondii*), responsible for a global parasitosis affecting a third of the worldwide population [19]. Since this article focuses on the new features brought to AMIDE and their consequences on general performances and workflow efficiency, the results presented hereafter will not yet describe the detailed analysis of the docking experiments.

The remainder of this paper is organized as follows. Section 2 compares the performance of the versions and the accuracy of the results. Section 3 presents our conclusions and future works. Finally, Section 4 presents the comparison methodology, our study case and the datasets preparation steps.

## 2. Results

### 2.1. Estimation of Performance Enhancement with AMIDE v2 Compared to AMIDE v1

AMIDE version performances were compared by computing the total calculation time of docking of the ligand (4) on the 12 docking boxes of the calcium-dependent protein kinase 1 from *T. gondii* (TgCDPK1, 6BFA ID in the Protein Data Bank). Three comparisons were made for 1, 10, and 100 runs, the other parameters being kept constant. For AMIDE v1, the grid preparation was also considered since it was part of the workflow process. For this comparison, the calculation processes with AMIDE v1 and AMIDE v2 were distributed on a 14 cores processor architecture and one GPU, respectively. In the case of AMIDE v1, all the cores were used to prepare the grids, and 12 cores were used for the 12 boxes docking.

**Figure 2:**
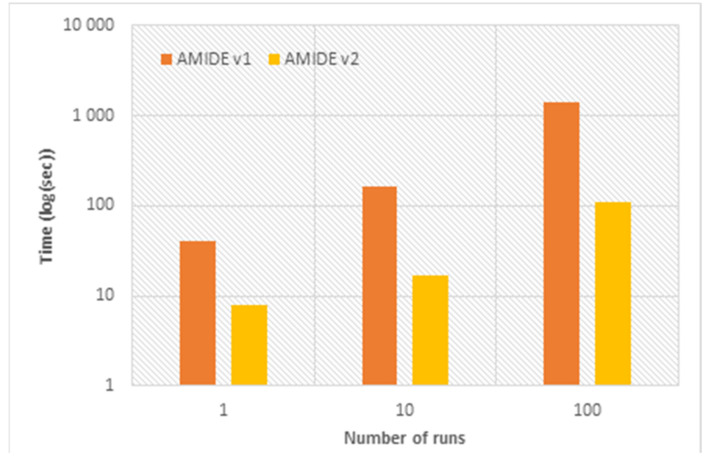
Performance comparison between the two AMIDE versions and depending on the number of docking runs. A log scale is used on the *y*-axis.

As highlighted in Figure 2, AMIDE v2 allowed a significant speed-up as compared to AMIDE v1. For a number of runs of 1, 10, and 100, the speed-up factors (defined as the ratio between the execution time of the AMIDE v1 and the execution time of the AMIDE v2) were 5.2, 9.7, and 12.4, respectively. This improvement in terms of calculation time will be of primary importance for massive inverse docking involving tens of proteins and thousands of ligands.

### 2.2. Inverse Docking with AMIDE v1 and AMIDE v2: Performance Enhancement Observed on the Toxoplasma gondii Dataset

**Figure 3:**
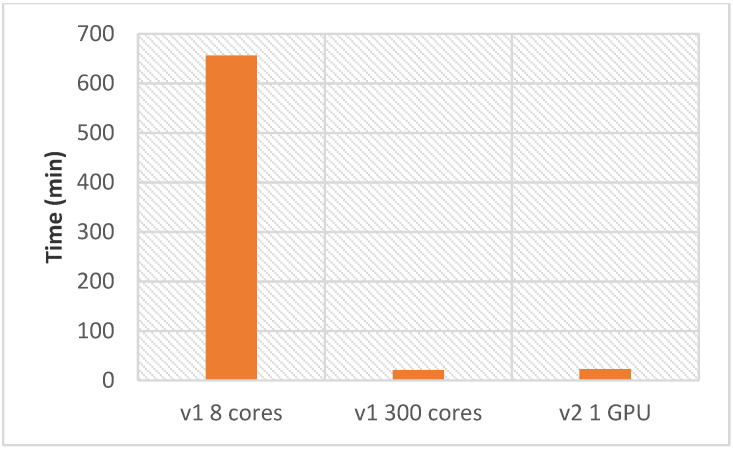
Comparison of the calculation time between the two AMIDE versions. Data were graphed for ligand (4) docked on the *T. gondii* dataset (87 proteins). The use of a personal PC was represented through the consideration of 8 cores and 1 GPU in the case of AMIDE v1 and AMIDE v2, respectively. The calculation time obtained with AMIDE v1 became as good as the one obtained with AMIDE v2 using 300 cores. The number of docking experiments per box was equal to 20.

We also compared the computation time for the whole inverse docking process with each AMIDE version for ligand (4) and the 87 proteins of the *T. gondii* dataset (Figure 3).

Using AMIDE v2 with a single GPU requires 23 min to perform the complete reverse docking process (one ligand and 87 proteins), while 300 cores are required to perform the same tasks as quickly on AMIDE version 1. As a comparison, a PC-like configuration (8 cores) led to a calculation time of 656 min.

### 2.3. Towards the Confrontation of Ligands and Proteins Databases: Performance of AMIDE v2 in High Throughput Inverse Screening

**Figure 4:**
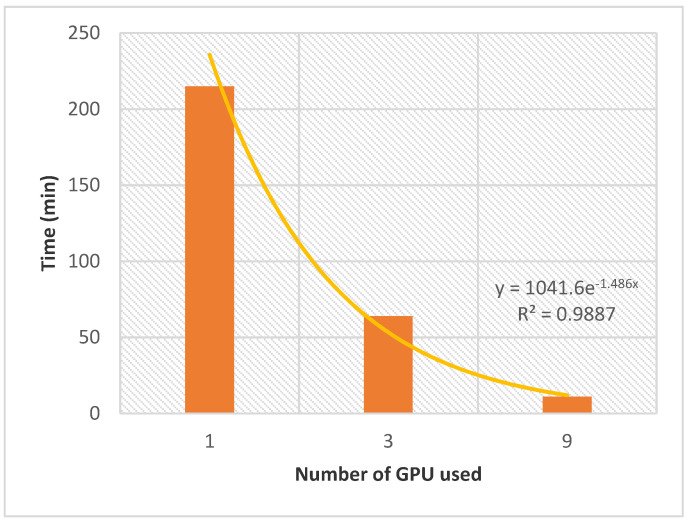
Evolution of the time needed to complete the double screening as a function of the number of GPUs. The experiments were realized with AMIDE v2, between the set of 9 ligands and the *T. gondii* dataset. The number of docking experiments per box was set to 20.

AMIDE v2 was evaluated in a double screening process between the *T. gondii* target dataset (87 proteins) and the database of nine ligands presented in Section 4 to evaluate the potential of the improved AMIDE version for further high throughput inverse screening applications involving tens of proteins and thousands of ligands. The evolution of the computation time with the number of GPUs is shown in Figure 4. Although the ligand database could be considered small, and the *T. gondii* dataset of average size, the whole process performed with AMIDE v2 generated a relatively significant number of output files (AutoDock DLG format). Indeed 9396 DLG files (12 boxes × 87 proteins × 9 ligands) were associated with 783 (9 ligands × 87 proteins) different protein-ligand docking experiments.

The allocation of a single GPU led to a calculation time of 215 min. The latter was divided by 3.4 and 19.5 when 3 and 9 GPUs were allocated, respectively. The exponential nature of the performance gain as a function of the number of allocated GPUs confirms the potential of AMIDE v2 to support in silico high throughput inverse screening.

Finally, to demonstrate the performance of the system, the 1018 compounds of the essential National Chemical Library (NCL) were submitted to the complete receptor database (87 proteins). A total of 50 GPUs was used, and it took 13 h and 30 min to AMIDE v2 to achieve the 88,566 dockings. A total of 1,062,792 docking files were generated (12 grids × 87 receptors × 1018 ligands) for 21,255,840 docking poses (1,062,792 dockings × 20 runs).

### 2.4. Analysis of High Throughput Screening

**Figure 5:**
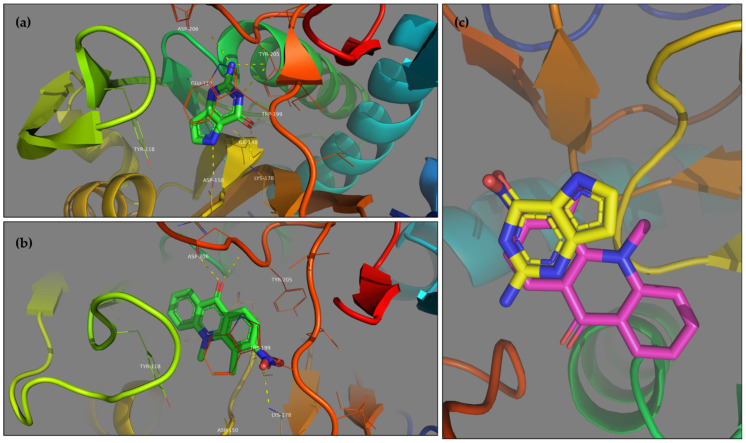
Comparison of the positions of the co-crystallized 9DG ligand (**a**) and the top ligand 393 (**b**) on 1FSG protein. Superposition of the poses of the co-crystallized 9DG ligand and the top ligand 393 (**c**). Both are placed in the same pocket and at least three hydrogen bonds (dashed yellow lines) are highlighted between ligands and residues LYS-178, TYR-205 and ASP-206. π-stacking is observed in the two cases for residue TRP-199.

The analyses of high throughput screening of the 1018 compounds of essential NCL against the 87 receptors of the dataset was performed and data analysis is presented hereafter. Table 1 shows the top ten results of the protein-ligand complex ranking. The free energies of binding and the population associated with these clusters indicate the high affinity of these ligands towards the considered targets. Further bioassays will be performed to investigate the in vitro activity of these compounds on *Toxoplasma gondii*. Nevertheless, the comparison of top ligand 393 and co-crystallized ligand 9DG on protein 1FSG (Figure 5) shows that they are co-localized in the binding site. Due to structure analogy, similar hydrogen bonds are highlighted as well as π-stacking interactions.

### 2.5. Pose Comparison Obtained with the Two AMIDE Versions

**Figure 6:**
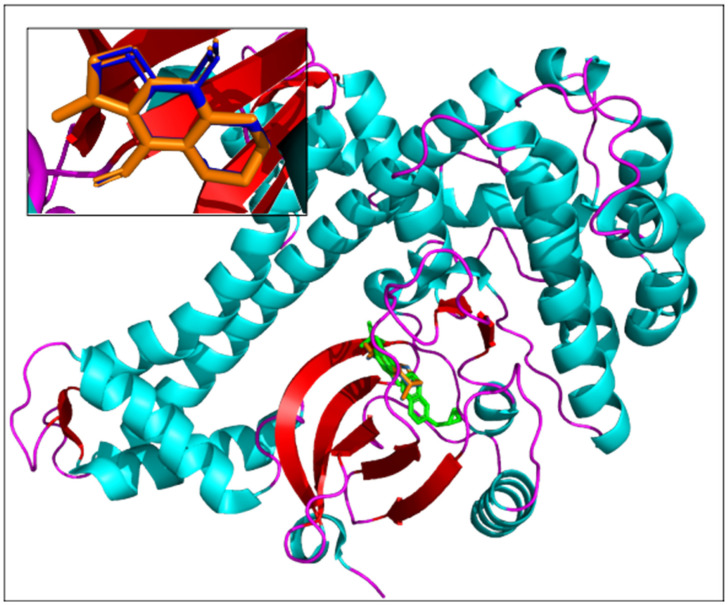
Comparison of experimental and docked poses of the protein associated with 6BFA PDB ID. The co-crystallized ligand is represented in green, and the best poses of ligand (7) are pictured in blue (AMIDE v1) and orange (AMIDE v2). The insert is a zoom-in representing the theoretical poses of ligand (7) in the binding site.

Execution time is a crucial parameter when massive in silico screening workflows are used for drug candidate discovery. The other determinant aspect of a docking approach is its reliability and ability to accurately predict the 3D conformation of a molecular complex. As a final validation step of the AMIDE v2 process, the results obtained for the complexes “ligand (7)/TgCDPK1 (PDB ID 6BFA)” and “co-crystallized ligand (UW5)/TgCDPK1 (PDB ID 6BFA)” were analysed. The poses corresponding to the most populated cluster with the lowest free energy of binding with AMIDE v1 and AMIDE v2 were compared and are shown in Figure 6. Cluster allocation was processed with an RMS tolerance of 2.0 Å between conformations. Figure 6 highlights that the best poses are in the co-crystallized ligand binding site, confirming the relevance of the complexes being compared.

The free energies of binding determined with AMIDE v1 and v2 were equal to −6.27 kcal/mol and −6.30 kcal/mol, respectively. The RMSD between the best poses proposed by AMIDE v1 and AMIDE v2 was equal to 0.106 Å. These results allow us to conclude that no significant differences exist between the docking results generated by the two versions of AMIDE, thus confirming the interest in using the new, improved version, especially for large sets of proteins and ligands.

## 3. Discussion and Conclusions

The aim of this article is not related to the analyses of docking results but rather to the enhancement of general performances and associated features. Thus, the conclusion and related discussion focus solely on the workflow improvements.

This new version of AMIDE drastically increases the speed of docking calculations. The combined use of a GPU version of AutoDock and the slicing in twelve grids of each protein allowing the distribution of calculations on multiple GPUs allow more than ever its use in reverse high-throughput screening. Cutting into 12 boxes is the opposite of a pocket search on proteins. This process ultimately can rapidly screen millions of ligands on dozens of proteins in record time without being limited to some regions of those proteins.

Contrary to the AMIDE v1 process, which suffered from performance reduction when increasing reserved resources, the AMIDE v2 process displayed better performances, since computation times ratios could be more than inversely proportional to allocated resources ratios. Increasing the number of CPUs (AMIDE v1) leads to high data exchange and impaired computation speed. In contrast, the use of GPU (AMIDE v2) decreased the data flow and thus led to better performances.

The local-search method (LSMET) initially embedded in AutoDock 4.2 was Solis-Wets. AMIDE also uses this version even if AutoDock-GPU is now proposed with LSMET ADADELTA [15]. The Solis-Wets method has been adapted to be run in parallel processing, leading to increased performances (especially by the reduction in calculation time). It has been shown that the modification of the algorithm did not alter the docking quality. Note that we have estimated that the use of Solis-Wets algorithm as LSMET from AutoDock-GPU should stay the standard for AMIDE because the new implementation of ADADELTA in AutoDock-GPU is still too recent and calculation times are slightly longer. That said, ADADELTA could be employed later on AMIDE since it can improve the docking results quality.

AMIDE was mainly used and intended for HPC. However, its use is also possible on a classic PC equipped or not with a GPU card. The new AMIDE version depends directly on AutoDock-GPU computing capabilities, therefore the parallelization of calculations in CPU version also exists but is less powerful than in GPU version. In GPU version, AutoDock-GPU made it possible to implement upper limits for docking parameters in order not to exceed the few GBs of memory used to match a maximum of common (on-chip) GPU cards. However, the use of an on-board graphics processor is still possible. In the case of personal computing, the acquisition of GPU hardware is relatively affordable compared to the use of several CPUs for equivalent computing capacities.

Although AMIDE was first designed for inverse virtual screening (one ligand against multiple targets), it is also possible to use it in simple virtual screening (multiple ligands against one specific target). The results presented in this study also demonstrated that AMIDE has a high potential to solve high throughput inverse screening (multiple ligands against multiple targets) issues. Future works will be focused on the implementation of artificial intelligence (AI) in the workflow to focus the search only on the most relevant areas of the proteins. AI could also be integrated in the post processing analyses as part of a decision support tool supplemented by molecular dynamic simulations.

## 4. Materials and Methods

### 4.1. From AMIDE v1 to AMIDE v2: Modifications and Improvements

AutoDock-GPU is an OpenCL-accelerated version of AutoDock 4.2.6 that is also CUDA compatible. It was advertised as capable of accelerating calculations up to 56x in Solis-Wets local search method [20]. More and more sectors such as scientific drills, financial simulations, and even AI in the broad sense have adopted GPGPU (General-Purpose computation on Graphics Processing Units) calculation. Computing under GPU is based on an embarrassingly parallel architecture consisting of grouping several hundred calculation units (cores). A GPU architecture is based on the SIMT (Single Instruction Multiple Thread) model, making it possible to launch parallel instances of a program on *n* different datasets. The AMIDE v2 workflow has been made compatible with GPU computing and can handle hundreds of calculations (see Figure 1) compared to mono-tasked CPU cores. This leads to a drastic decrease in input/output errors by limiting data flow overload. The amount of IT resources is then reduced, and workload manager user limitations are lowered.

The management of ligand inputs/outputs, as well as the respective association files (temporary and result files), were also optimized to favor the use of GPU acceleration. A configuration script was created to facilitate AMIDE installation and deployment. In addition, a specific python version and associated packages were also embedded so as not to be confronted with dependency or incompatibility errors.

Pre-docking files’ (grids, atom map) generation time was also highly reduced. One file was generated for each ligand and each protein grid, while universal (i.e., the grid parameters for all available atom types) pre-docking files are now generated once for the whole dataset.

A script was written in bash to automatically submit ligand and protein file lists to AutoDock GPU, preventing unsolicited errors during AMIDE calculations due to input-output conflicts. This also means that large libraries of ligands can be screened automatically.

Another consequence of having a unique submission list is that it allows the use of a SLURM Job Array. A job refers to a list of dockings to be performed and addressed to the requested resources. In this way, the queue manager is not slowed down, and input/output communications do not impact the AMIDE framework’s general performances.

Because AMIDE uses AutoDock, certain limitations, such as the constraint introduced on certain types of ligands, have always to be considered. The extensive list of AMIDE v2 compatible atom types is available in Table 2.

### 4.2. Calculations

The v2018 supercomputer at URCA ROMEO Datacenter is based on a Sequana X1000 type solution (ATOS). Its equipment includes 115 servers composed of Intel^®^ Skylake 6132 2.6 GHz processors totaling 3,220 cores and 280 accelerators Nvidia P100 SXM2. The running SLURM daemon is version 16.05.11-Bull.1.3, running under RedHat 7.4 Maipo and using CUDA 10.0 and gcc 7.4.0.

### 4.3. Receptors Dataset

The receptors set was generated from a list containing approximately 200 PDB structures from Protein Data Bank (PDB). A functional dataset of 87 structures was obtained by selecting only X-ray structures with resolution less than 3 Å. Water and other crystallographic residues were removed. Only chains of interest were preserved according to global stoichiometry. When necessary, apo-forms were generated from co factor-forms such as NAD or AMP. Three proteins (PDB identifier 4OKR, 1QK5, 1DGM) include one mutation compared to the reference sequence, and two mutations are present for the protein 4A5B. No energy minimization was performed.

**Figure 7:**
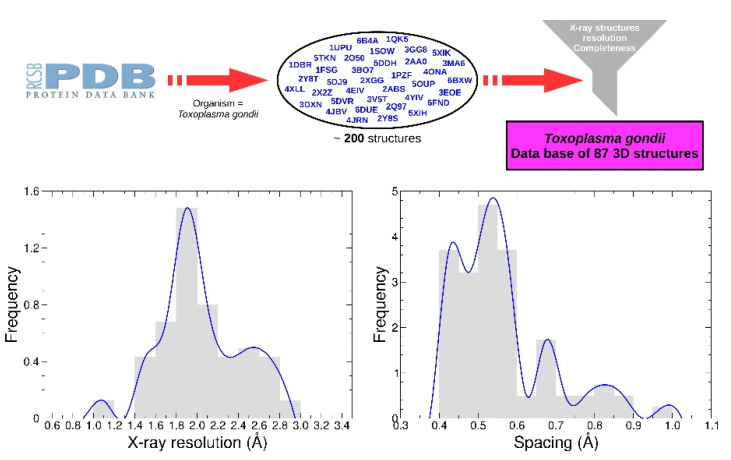
*T. gondii* protein selection methodology. Protein structures were obtained from the Protein Data Bank. About 200 structures of *T. gondii* proteins were selected and then filtered according to their acquisition method (X-ray), resolution (less than 3 angstroms), and completeness. The final dataset contains 87 structures whose resolution (graph on the left) and spacing (graph on the right) distribution are presented.

Polar hydrogen atoms were added prior to grid calculations, and the receptors were divided into 12 boxes with the AMIDE dataset preparation function. Depending on the protein’s size, the spacing used to compute the grids ranged from 0.40 to 1.00 Å (see Figure 7). All associated data are available in supplementary data (Table S1).

### 4.4. Ligands Dataset

**Figure 8:**
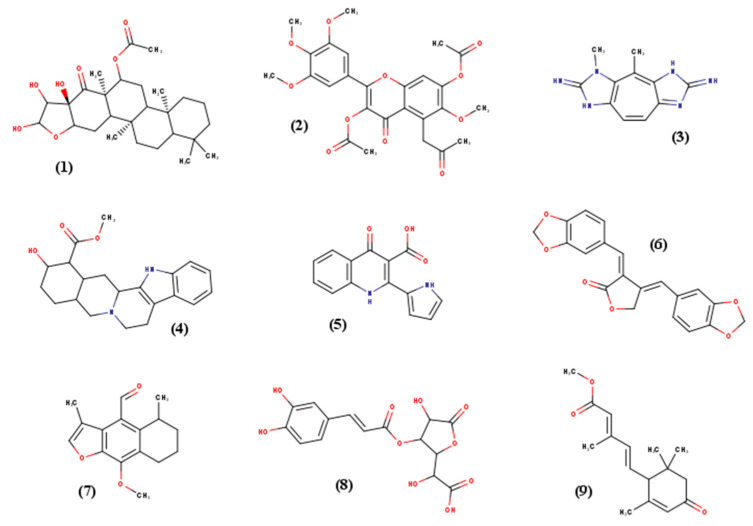
2D representation of the nine ligands used in this study. These schemes illustrate the diversity in geometry as well as in the chemical functions.

Nine small molecules were selected for this study according to their various chemical classes (atom type, structure) and torsional degrees of freedom (TORSDOF). They were chosen from PNMRNP (Predicted carbon-13 NMR data of Natural Products), a curated and annotated small natural molecules database [21]. All the relevant characteristics are presented in Table 3, and 2D structures are available in Figure 8. For high-throughput screening assay, French essential National Chemical Library (essential NCL) (https://chembiofrance.cn.cnrs.fr/en/composante/chimiotheque (accessed on 5 April 2021)) was used. The NCL presents more than 75,000 compounds of natural, synthetic or hemisynthetic origin deposited by French academic partner laboratories. The essential NCL represents the chemical diversity of this database through 1040 compounds. These are the ones that have been used here. Within this database, 22 compounds were identified as not compatible with AMIDE because of TORSDOF too high or atypic atom types. Thus, 1018 compounds of essential NCL were used.

### 4.5. Ligands Preparation

Ligands must be prepared according to AutoDock-GPU requirements. The output must be a 3D representation in PDBQT format. The first step was to convert a 2D structure of any format into a 3D structure. This was done using Schrödinger LigPrep 2020-3 software (LigPrep, Schrödinger, LLC, New York, NY, USA) with OPLS3e as force filed, ionization ah pH 7, salt removal and without tautomer generation. Other tools such as RDKit (https://rdkit.org/) or Avogadro (https://avogadro.cc/) could also be used. The second step was to convert SDF output files from LigPrep into PDBQT format files. This was done with Open Babel 3.1.1 [22]. Finally, AMIDE compatibility verification tool (TORSDOF < 32 and compatible atom type) was used for ligand validation.

### 4.6. Grid Preparation

Grid parameter files required for the use of AutoDock 4.2 (generated by AutoGrid 4) were generated for each ligand with AMIDE v1, which was time-consuming and tedious. Moreover, for a given atom type, the files were the same from one ligand to another one since the geometric definition (size and number of points) of the grid was strictly protein-dependent. Henceforth, with the upgraded AMIDE protocol, the grid generation is performed within a single unified preliminary step. The implemented strategy consists of creating a dummy ligand that could be described as a universal ligand that contains every AutoDock compatible atom type. During the docking step, AMIDE v2 uses only the necessary grid parameter files associated to the considered “real” ligand. Table 2 presents the list of all atom type available in AMIDE.

### 4.7. High Throughput Screening Analyses

The first step was to perform clustering of docking poses of each ligand-receptor complex. A modified version of AutoDock Tools was used, and clustering was performed using radius of gyration. Each identified cluster was then scored using Equation (2) (scoring 1) where *dG_min_* is the minimal free energy of binding identified in the complex, *dG_x_* the free energy of binding of the considered cluster, *Pop_max_* the maximum cluster population identified in the complex and *Pop_x_* the population of the considered cluster.

(2) $$Scoring\;1=\sqrt{\frac{\left|dG_{min}-dG_{x}\right|}{\left|dG_{min}\right|}+\frac{\left|Pop_{max}-Pop_{x}\right|}{\left|Pop_{max}\right|}}$$

(3) $$Scoring\;2=\sqrt{\frac{\left|dG_{min}-dG_{x}\right|}{\left|dG_{min}\right|}+\frac{\left|Pop_{max}-Pop_{x}\right|}{\left|Pop_{max}\right|}+\frac{\left|Score\;1_{min}-Score\;1_{x}\right|}{\left|Score\;1_{min}\right|}}$$

The clusters were then sorted according to their rank. The lower the scoring, the better the cluster. All clusters of all ligand-receptor complexes were then compared and re-ranked using the scoring function presented in Equation (3) (*scoring* 2). This function is similar with *scoring* 1 and includes an additional scoring term, where Score 1*_min_* is the score corresponding to the best ligand-receptor complex among all the clusters. Thanks to this method, there is no arbitrary threshold definition. Visualization of poses was performed using PyMol 2 (The PyMOL Molecular Graphics System, Version 2.0 Schrödinger, LLC).

## Figures and Tables

**Table 1:** Top ten protein–ligand complexes from the virtual screening of the 1018 compounds of essential NCL against 87 *Toxoplasma gondii* receptors. a.u. stands for arbitrary unit.

Protein	Ligand	POP	dG (kcal/mol)	Score 1 (a.u.)	Score 2 (a.u.)
1FSG	393	159	−9.42	0.3375	0.3425
3OTR	369	158	−9.11	0.3416	0.3537
1FSG	32	159	−8.41	0.3375	0.3729
1FSG	123	158	−8.47	0.3416	0.3748
2I44	344	159	−8.30	0.3375	0.3777
3Q5Z	393	156	−8.55	0.3500	0.3818
1FSG	460	157	−8.38	0.3458	0.3833
1FSG	439	159	−7.99	0.3375	0.3926
3STH	671	155	−8.32	0.3541	0.3965
1FSG	234	157	−8.08	0.3458	0.3969

**Table 2:** Atom type available for AMIDE based on AutoDock 4.2.6.

Name	Atom Type	Name	Atom type
**H**	Non H-bonding Hydrogen	**S**	Non H-bonding Sulphur
**HD**	Donor 1 H-bond Hydrogen	**Cl or CL**	Non H-bonding Chlorine
**HS**	Donor S Spherical Hydrogen		
**C**	Non H-bonding Aliphatic Carbon	**Ca or CA**	Non H-bonding Calcium
**A**	Non H-bonding Aromatic Carbon		
**N**	Non H-bonding Nitrogen	**Mn or MN**	Non H-bonding Manganese
**NA**	Acceptor 1 H-bond Nitrogen		
**NS**	Acceptor S Spherical Nitrogen	**Fe or FE**	Non H-bonding Iron
**OA**	Acceptor 2 H-bonds Oxygen		
**OS**	Acceptor S Spherical Oxygen	**Zn or ZN**	Non H-bonding Zinc
**F**	Non H-bonding Fluorine		
**Mg** **or MG**	Non H-bonding Magnesium	**Br or BR**	Non H-bonding Bromine
**P**	Non H-bonding Phosphorus	**I**	Non H-bonding Iodine
**SA**	Acceptor 2 H-bonds Sulphur		

**Table 3:** Presentation of the dataset of the nine ligands used in this study with associated molecular formula and TORSDOF. This last parameter represents the intrinsic flexibility of the ligands as considered within the docking process of AutoDock.

ID	Molecular Formula	TORSDOF
(1)	C_27_H_42_O_7_	5
(2)	C_25_H_24_O_2_	11
(3)	C_11_H_12_N_6_	0
(4)	C_21_H_26_N_2_O_3_	3
(5)	C_14_H_10_N_2_O_3_	2
(6)	C_20_H_14_O_6_	2
(7)	C_16_H_18_O_3_	2
(8)	C_15_H_14_O_10_	10
(9)	C_16_H_22_O_3_	4

## Data Availability

Full documentation of the AMIDE framework will be available soon on our Gitlab instance. Code is available on request from the corresponding author.

## Supplementary Materials

The following are available online at https://www.mdpi.com/article/10.3390/ijms22147489/s1.
