# Supplementary material for: AMIDE v2: High-Throughput Screening Based on AutoDock-GPU and Improved Workflow Leading to Better Performance and Reliability
Source: Int J Mol Sci. 2021 Jul 13;22(14):7489. doi: 10.3390/ijms22147489 (PMC8307493; doi:10.3390/ijms22147489)
Supplement: Supplementary file 1 [file ijms-22-07489-s001.zip › ijms-1262009-supplementary.pdf]

International Journal of *Molecular Sciences*

MDPI

Supplementary data

Table S1 : *Toxoplasma gondii* protein dataset

Protein	PDB	Uniprot	Reso-lution (Å)	Global Stoi-chiometry	Chain(s)
ACYL CARRIER PROTEIN	2QNW	O77470	1.90	Monomer	A
ADENOSINE KINASE + ADenosiNe	1DGM	Q9TVW2	1.80	Monomer	A
ADENOSINE KINASE FROM 1DGM	1DGM	Q9TVW2	1.80	Monomer	A
APICAL COMPLEX LYSINE METHYLTRANSFER-ASE	6FND	B9Q0K5	2.10	Homo 2-mer	B / C
APICAL MEMBRANE ANTIGEN AMA1	4YIV	S8GKS3	1.93	Monomer	A
BIFUNCTIONAL DIHYDROFOLATE REDUC-TASE-THYMIDYLATE SYNTHASE	6AOI	Q07422	2.97	Monomer	A
BRADYZOITE PSEUDOKINASE 1	6M7Z	A0A452CSY6	2.50	Monomer	A
BRADYZOITE SURFACE ANTIGEN BSR4	2JKS	Q95NL6	1.90	Homo 3-mer	
CALCIUM-DEPENDENT PROTEIN KINASE 3	3HZT	Q3HNM6	2.00	Homo 4-mer	A / B / C / D
CALMODULIN-DOMAIN PROTEIN KINASE 1	6BFA	A0A0F7UUA6	2.80	Monomer	B
CALMODULIN-DOMAIN PROTEIN KINASE 1	3I79	Q9BJF5	2.04	Monomer	A
CALMODULIN-DOMAIN PROTEIN KINASE 1 IN-ACTIVATED FORM + ANP	3KU2	Q9BJF5	2.30	Monomer	A
CATHEPSIN L PROTEASE	3F75	Q6DMN0	1.99	Homo 2-mer	A / B
CYCLOPHILIN	3BKP	D0VWS4	1.80	Monomer	A
DEOXYRIBOSE-PHOSPHATE ALDOLASE	4EIV	B9PVB4	1.37	Monomer	A
DJ-1 FAMILY PROTEIN	4XLL	B9PZH8	2.08	Homo 2-mer	A / B
DOUBLECORTIN	6B4A	B6KAS6	2.00	Homo 4-mer	A / B / C / D
EGF FAMILY DOMAIN-CONTAINING PROTEIN	4Z81	S7UIL3	2.05	Homo 2-mer	A / B
ENOLASE	3OTR	B9PH47	2.75	Homo 2-mer	A / B
ENOYL-ACYL CARRIER REDUCTASE	2O50	Q6UCJ9	2.90	Homo 2-mer	A / B
ENOYL-ACYL CARRIER REDUCTASE	3NJ8	Q6UCJ9	2.70	Homo 6-mer	A / B / C / E / F / G
ENOYL-ACYL CARRIER REDUCTASE	4O1M	Q6UCJ9	2.00	Monomer	A
ENOYL-ACYL CARRIER REDUCTASE + NAD	2O2S	Q6UCJ9	2.60	Hetero 2-mer	B
FRUCTOSE-1 /6-BISPHOSPHATE ALDOLASE	4TU1	B9PW35	2.00	Monomer	A
FRUCTOSE-BISPHOSPHATE ALDOLASE	5TJS	Q8I8I2	1.78	Monomer	A
FRUCTOSE-BISPHOSPHATE ALDOLASE + SCHIFF	5TKP	Q8I8I2	2.09	Hetero 2-mer	A
GLUCOSE-6-PHOSPHATE ISOMERASE	3UJH	NA	2.10	Hetero 2-mer	A
GLUTAMINE AMINOACYL-TRNA SYNTHETASE	4P2B	B6KTZ0	2.80	Monomer	A
GLYCERALDEHYDE-3-PHOSPHATE DEHYDRO-GENASE + NAD	3STH	Q9BKE2	2.25	Homo 2-mer	A / B

GLYCERALDEHYDE-3-PHOSPHATE DEHYDRO-GENASE FROM 3STH	3STH	Q9BKE2	2.25	Homo 2-mer	A / B
HYPOXANTHINE-GUANINE PHOSPHORIBOSYL-TRANSFERASE	1FSG	Q26997	1.05	Homo 2-mer	A / C
HYPOXANTHINE-GUANINE PHOSPHORIBOSYL-TRANSFERASE + GMP	1QK3	Q26997	1.65	Monomer	E
HYPOXANTHINE-GUANINE PHOSPHORIBOSYL-TRANSFERASE + IMP	1QK4	Q26997	1.90	Monomer	A
HYPOXANTHINE-GUANINE PHOSPHORIBOSYL-TRANSFERASE + XMP	1QK5	Q26997	1.60	Monomer	A
IMC SUB-COMPARTMENT PROTEIN ISP1	4CHM	B9PKK4	2.10	Monomer	A
IMC SUB-COMPARTMENT PROTEIN ISP3	4CHJ	B9PJE6	2.32	Monomer	A
INFLAMMATORY PROFILIN	3NEC	Q58NA1	1.70	Homo 2-mer	A / B
LACTATE DEHYDROGENASE APO	1PZE	P90613	1.95	Homo 4-mer	
LACTATE DEHYDROGENASE WITH APAD	1PZG	P90613	1.60	Homo 4-mer	A / B / C / D
LACTATE DEHYDROGENASE WITH NAD	1PZH	P90613	1.90	Homo 4-mer	A / B / C / D
L-LACTATE DEHYDROGENASE + NAD	1SOW	Q27797	1.90	Monomer	A
L-LACTATE DEHYDROGENASE APO	1SOV	Q27797	1.90	Homo 2-mer	A / B
MAJOR SURFACE ANTIGEN P30	1KZQ	P13664	1.70	Homo 2-mer	A / B
MICRONEMAL PROTEIN 1	2JH1	O00834	1.90	Homo 4-mer	
MICRONEMAL PROTEIN MIC2	4OKR	O00816	2.60	Homo 4-mer	A / B / C / D
MICRONEME PROTEIN 2	2XGG	NA	2.05	Homo 4-mer	A / B / C / D
MIF	4DH4	A1XDS9	1.82	Homo 4-mer	
MITOCHONDRIAL ASSOCIATION FACTOR 1	6BXR	A0A140H546	1.60	Homo 4-mer	A / B / C / D
MITOCHONDRIAL ASSOCIATION FACTOR 1 + APR	6BXW	A0A140H546	1.65	Homo 4-mer	A / B / C / D
MITOGEN-ACTIVATED PROTEIN KINASE	3RP9	B6KP12	2.40	Monomer	B
MYOSIN A	6DUE	S7W634	2.60	Homo 4-mer	
MYOSIN A FROM 6DUE	6DUE	S7W634	2.60	Homo 4-mer	
MYOSIN LIGHT CHAIN TGMLC1	5VT9	Q95UJ7	1.85	Homo 4-mer	A / B / C / D
NUCLEOSIDE DIPHOSPHATE KINASE	5BXI	B9Q287	1.70	Homo 4-mer	A / B / C / D
NUCLEOSIDE-TRIPHOSPHATASE 1	4A57	Q27893	2.00	Homo 2-mer	A / B
NUCLEOSIDE-TRIPHOSPHATASE 1 + AMP	4A59	Q27893	2.20	Homo 6-mer	
NUCLEOSIDE-TRIPHOSPHATASE 2	4A5B	Q27895	2.48	Monomer	A
ORNITHINE AMINOTRANSFERASE / MITO-CHONDRIAL / PUTATIVE	5EAV	S8EY38	1.60	Homo 4-mer	
OROTIDINE 5'-PHOSPHATE DECARBOXYLASE	4MJZ	Q12339	2.75	Monomer	A

PEPTIDYL-PROLYL CIS-TRANS ISOMERASE CYCLOPHILIN-TYPE	3BO7	D0VWS5	2.35	Homo 2-mer	A / B
PERFORIN-LIKE PROTEIN 1	6D7A	A1E348	1.13	Monomer	B
PERFORIN-LIKE PROTEIN 1	5OUP	V5BCL0	2.03	Hetero 2-mer	A / P
PHOSPHOGLYCERATE MUTASE PGMII	4ODI	S8GJT7	2.60	Homo 4-mer	
PROLYL-TRNA SYNTHETASE (PRORS)	5XIF	S8G8I1	2.48	Homo 4-mer	
PROTEIN KINASE A	5J3U	A0A0F7V8B2	1.80	Homo 4-mer	
PROTEIN KINASE A + AMPc	5J3U	A0A0F7V8B2	1.80	Homo 4-mer	A / B / C / E
PROTEIN PHOSPHATASE 2C	3D8K	A4GX63	2.71	Homo 4-mer	
PTERIN-4A-CARBINOLAMINE DEHYDRATASE	2V6U	Q2Q449	1.6	Homo 4-mer	
PURINE NUCLEOSIDE PHOSPHORYLASE	3MB8	Q2HXR2	1.90	Monomer	A
PYRUVATE KINASE	3EOE	Q969A2	2.31	Monomer	A
RHOPTRY KINASE 2 + 8	3BYV	O15693	1.80	Hetero 2-mer	B / D
RHOPTRY KINASE DOMAIN	3DZO	Q06AK3	1.80	Homo 4-mer	A / B / C / D
RHOPTRY KINASE FAMILY PROTEIN	4JRN	Q2PAY2	2.71	Monomer	A
RHOPTRY PROTEIN 5 C + GDP + ADP	4LV8	I6ZQR7	1.72	Monomer	A
RHOPTRY PROTEIN 5 C FROM PDB 4LV8	4LV8	I6ZQR7	1.72	Homo 2-mer	A / D
RIBULOSE 5-PHOSPHATE ISOMERASE	4NML	S8GQK2	2.60	Homo 2-mer	A / D
RIBULOSE-PHOSPHATE 3-EPIMERASE	4NU7	NA	2.05	Monomer	A
ROP2	2W1Z	Q27007	1.97	Monomer	A
ROP5B	3Q5Z	F2YGR7	1.90	Monomer	A
ROP5B + ATP	3Q60	F2YGR7	1.72	Monomer	A
SEDOHEPTULOSE-1 /7 BISPHOSPHATASE / PUTATIVE	4IR8	B9PW60	1.85	Monomer	A
SERINE-THREONINE PHOSPHATASE 2C	2I44	Q8WPN9	2.04	Homo 2-mer	A / B
SOLUBLE INORGANIC PYROPHOSPHATASE	5WRT	Q4VUZ3	2.35	Homo 2-mer	A / B
TRIOSEPHOSPHATE ISOMERASE	5UPR	A0A125YP67	2.00	Homo 4-mer	A / B / C / D
UBIQUITIN-CONJUGATING ENZYME E2	2AYV	NA	2.00	Monomer	A
URACIL PHOSPHORIBOSYLTRANSFERASE + URA	1BD4	Q26998	2.20	Monomer	A
URACIL PHOSPHORIBOSYLTRANSFERASE FROM 1BD4	1BD4	Q26998	2.20	Homo 7-mer	
